# B Cell Receptor Repertoire Analysis in Malaria-Naive and Malaria-Experienced Individuals Reveals Unique Characteristics of Atypical Memory B Cells

**DOI:** 10.1128/mSphere.00726-21

**Published:** 2021-09-15

**Authors:** Ashley E. Braddom, Sebastiaan Bol, S. Jake Gonzales, Raphael A. Reyes, Kenneth Musinguzi, Felistas Nankya, Isaac Ssewanyana, Bryan Greenhouse, Evelien M. Bunnik

**Affiliations:** a Department of Microbiology, Immunology and Molecular Genetics, Long School of Medicine, The University of Texas Health Science Center at San Antonio, San Antonio, Texas, USA; b Infectious Disease Research Collaboration, Kampala, Uganda; c London School of Hygiene and Tropical Medicine, London, UK; d Department of Medicine, University of California San Francisco, San Francisco, California, USA; University at Buffalo

**Keywords:** *Plasmodium*, adaptive immune response, humoral immunity, IgM, HCDR3, somatic hypermutation

## Abstract

Malaria, caused by parasites of the *Plasmodium* genus, is responsible for significant morbidity and mortality globally. Chronic Plasmodium falciparum exposure affects the B cell compartment, leading to the accumulation of atypical memory B cells (atMBCs). IgM-positive (IgM^+^) and IgG^+^ atMBCs have not been compared in-depth in the context of malaria, nor is it known if atMBCs in malaria-experienced individuals are different from phenotypically similar B cells in individuals with no known history of *Plasmodium* exposure. To address these questions, we characterized the B cell receptor (BCR) repertoire of naive B cells (NBCs), IgM^+^ and IgG^+^ classical MBCs (cMBCs), and IgM^+^ and IgG^+^ atMBCs from 13 malaria-naive American adults and 7 malaria-experienced Ugandan adults. Our results demonstrate that P. falciparum exposure mainly drives changes in atMBCs. In comparison to malaria-naive adults, the BCR repertoire of *Plasmodium-*exposed adults showed increased levels of somatic hypermutation in the heavy chain V region in IgM^+^ and IgG^+^ atMBCs, shorter heavy chain complementarity-determining region 3 (HCDR3) in IgG^+^ atMBCs, and increased usage of IGHV3-73 in IgG^+^ cMBCs and both IgM^+^ and IgG^+^ atMBCs. Irrespective of *Plasmodium* exposure, IgM^+^ atMBCs closely resembled NBCs, while IgG^+^ atMBCs resembled IgG^+^ cMBCs. Physicochemical properties of the HCDR3 seemed to be intrinsic to cell type and independent of malaria experience. The resemblance between atMBCs from *Plasmodium*-exposed and naive adults suggests similar differentiation pathways regardless of chronic antigen exposure. Moreover, these data demonstrate that IgM^+^ and IgG^+^ atMBCs are distinct populations that should be considered separately in future analyses.

**IMPORTANCE** Malaria, caused by *Plasmodium* parasites, still contributes to a high global burden of disease, mainly in children under 5 years of age. Chronic and recurrent *Plasmodium* infections affect the development of B cell memory against the parasite and promote the accumulation of atypical memory B cells (atMBCs), which have an unclear function in the immune response. Understanding where these cells originate from and whether they are beneficial in the immune response to *Plasmodium* will help inform vaccination development efforts. We found differences in B cell receptor (BCR) properties of atMBCs between malaria-naive and malaria-experienced adults that are suggestive of divergent selection processes, resulting in more somatic hypermutation and differential immunoglobulin heavy chain V (IGHV) gene usage. Despite these differences, atMBCs from malaria-naive and malaria-experienced adults also showed many similarities in BCR characteristics, such as physicochemical properties of the HCDR3 region, suggesting that atMBCs undergo similar differentiation pathways in response to different pathogens. Our study provides new insights into the effects of malaria experience on the B cell compartment and the relationships between atMBCs and other B cell populations.

## INTRODUCTION

Malaria is a deadly disease mainly affecting children in sub-Saharan Africa and Southeast Asia. In 2019, there were an estimated 229 million cases globally caused predominantly by the parasites Plasmodium falciparum and Plasmodium vivax (1). Immunity to *Plasmodium* is slow to develop, requiring years of repeated exposures to the parasite to reach sufficient levels. The immune response is never sterilizing but, instead, provides protection against clinical disease. The factors contributing to this delayed response are incompletely understood, although it has become clear that chronic exposure to parasite antigens affects the memory B cell (MBC) compartment (2, 3).

Chronic disease settings, including chronic or recurrent infections and autoimmunity, drive the accumulation of a population of atypical MBCs (atMBCs) (4–8). In malaria-experienced individuals, atMBCs commonly represent 10 to 20% of all mature circulating B cells but can account for up to 30%, while in malaria-naive individuals, atMBCs usually make up less than 5% of this compartment (2, 3, 9, 10). In the malaria field, these cells are typically classified as CD19-positive (CD19^+^) CD21-negative (CD21^−^) CD27^−^ B cells, but this population frequently displays altered expression of other surface markers, including CXCR3, CXCR5, and CD11c, as well as the transcription factor T-bet (10–12). In the context of malaria, atMBCs also express inhibitory receptors, including FcRL5 (10–12). Upon P. falciparum infection, atMBCs show a peak in expansion around 10 days after malaria diagnosis and this cell population gradually contracts to background levels following clearance of infection (13, 14).

The origin and function of atMBCs are incompletely understood (15). It appears that the immune environment generated during a *Plasmodium* infection can drive the expansion of these cells, potentially by promoting the development of Th1-polarized T follicular helper (Tfh1) cells (16). It is thought that the interaction of B cells with Tfh1 cells in the B cell follicle results in bypassing the germinal center, leading to the generation of atMBCs (16). In addition, *in vitro* studies have demonstrated that atMBC-like cells can be generated from naive B cells (NBCs) in the presence of interferon gamma (IFN-γ) signaling, B cell receptor (BCR) cross-linking, and Toll-like receptor (TLR) 7/9 engagement (17, 18). AtMBCs share phenotypic and transcriptional similarities with an extrafollicularly activated CD21^−^ CD27^−^ B cell population expanded in individuals with systemic lupus erythematosus (SLE) and severe COVID patients, which suggests that atMBCs may be, in part, derived from extrafollicular activation of NBCs (19–21). In these settings, the abundance of class-switched atMBCs, also termed DN2 cells, is associated with disease severity (19, 21). The distinction between unswitched and class-switched CD21^−^ CD27^−^ B cells is likely to be important, as these cells may have different origins and functions. To aid in this distinction, it has been proposed that only class-switched CD21^−^ CD27^−^ B cells should be classified as atMBCs, while unswitched (IgM^+^/IgD^+^) CD21^−^ CD27^−^ B cells have been defined as activated NBCs (actNBCs) (22, 23).

Each B cell expresses a B cell receptor (BCR) that is used to bind to antigen. The BCR repertoire of a B cell subset captures the collection of BCRs expressed by that population of cells. This repertoire can be used to understand general characteristics of B cell subsets and provide insight into their developmental pathways and the stimuli driving B cell selection. In other chronic disease settings, repertoire analysis has shed light on the impact of infection on B cell populations and antibody responses. In the context of human immunodeficiency virus (HIV) infections, studying monoclonal antibody immunoglobulin heavy chain V (IGHV) gene usage, heavy chain complementarity-determining region 3 (HCDR3) length, and somatic hypermutation (SHM) levels has shown that persistent infection drives the usage of specific IGHV genes, long HCDR3s, and high levels of SHM compared to antibodies derived from acute infection settings (24). Furthermore, the IGHV4-34 gene, which is associated with autoreactivity (25, 26), was overrepresented among IgG^+^ classical memory B cells (cMBCs) in HIV-infected individuals with broadly neutralizing antibodies (27). These results suggest that certain BCR characteristics are selected during chronic infection and provide insight into the rules that govern the development of protective immune responses.

Malaria-associated IgG^+^ atMBCs and cMBCs were reported to have similar levels of SHM and similar IGHV gene usage (10, 28), suggesting that the factors that drive the differentiation of these B cell subsets do not select for specific BCR characteristics. However, the BCR repertoires of IgM^+^ and IgG^+^ atMBCs have not been compared. In addition, it is unknown whether atMBCs in malaria-experienced individuals are functionally different from phenotypically similar B cell subsets in unexposed individuals.

Here, we characterize the BCR repertoire of B cell subsets in the context of *Plasmodium* infection to uncover potential effects of malaria on the B cell compartment and to understand whether malaria-associated atMBCs have unique features that set them apart from phenotypically similar cells in nonexposed individuals. To this end, we compared the BCR repertoires of NBCs, cMBCs, and atMBCs from malaria-naive U.S. donors and malaria-experienced Ugandan donors. Our results demonstrate that P. falciparum exposure drives changes in the B cell compartment, predominantly in atMBCs, and that these changes are most likely driven by antigen selection. However, similarities in physicochemical properties of the HCDR3 regions among B cell subsets between malaria-naive and malaria-experienced individuals point toward similar differentiation pathways of malaria-associated atMBCs and atMBCs found in malaria-naive individuals.

## RESULTS

### Generation of B cell receptor sequencing data sets.

To study the effect of P. falciparum exposure on the B cell compartment, we performed BCR sequencing for seven malaria-experienced donors from Uganda and 13 malaria-naive donors from the United States (Table S1 [all supplemental material for this article can be found at https://doi.org/10.6084/m9.figshare.16449858.v3]). Among the malaria-naive U.S. donors were seven Black (US-B1 to US-B7) and six White (US-W1 to US-W6) adults, who were selected to rule out race as a variable in our analyses. The malaria-experienced donors were three healthy adult blood donors (UG-1 to UG-3) from a high-transmission region with presumed immunity against malaria, as is typical for adults from this region, and four participants of the PRISM ICEMR cohort (UG-4 to UG-7) (29). Medical history was not available for the three healthy blood donors, but high antibody titers against various malaria antigens confirmed frequent exposure to P. falciparum (Fig. S1 at the URL mentioned above). Medical history for participants of the PRISM ICEMR cohort is available at https://www.clinepidb.org.

For each donor, three B cell populations were fluorescence-activated cell sorter (FACS) sorted based on the expression of cell surface markers, naive B cells (NBCs; CD19^+^ CD21^+^ CD27^−^), classical memory B cells (cMBCs; CD19^+^ CD21^+^ CD27^+^), and atypical memory B cells (atMBCs; CD19^+^ CD21^−^ CD27^−^) (Fig. S2 at https://doi.org/10.6084/m9.figshare.16449858.v3). Sequence libraries of the heavy chain variable regions were generated with a template-switching PCR using unique molecular identifiers (UMI) to be able to correct for biases and errors introduced during PCR amplification and sequencing. Due to low cell input, the generation of atMBC libraries was unsuccessful for three malaria-naive donors (US-W3, US-W4, and US-W5). Additionally, we did not collect NBCs from three malaria-naive donors (US-B6, US-B7, and US-W6) and four malaria-experienced donors (UG-4, UG-5, UG-6, and UG-7).

Sequences supported by at least 6 reads with the same UMI were included in downstream analyses. The resulting UMI groups had average numbers of reads per UMI ranging from 11.6 to 298.8, indicating sufficient sequence depth for all samples (Tables S2 to S4 at https://doi.org/10.6084/m9.figshare.16449858.v3). IgM and IgG sequence reads were extracted based on unique sequences in the μ and γ constant regions. Since IgM and IgG antibodies are associated with protection from clinical malaria (30–33), we restricted our analysis to these isotypes. Reads were analyzed using the ImmCantation Portal (34). Three IgG^+^ atMBC samples were excluded from further analysis because we obtained less than 100 sequences per sample (Table S4 at the URL mentioned above). For five samples, we generated biological replicates of sequencing libraries to test the robustness of our library preparation protocol. Replicates were highly similar (Pearson’s *R*, 0.80 to 0.99) and were combined for downstream analysis (Fig. S3 at the URL mentioned above).

### IgM^+^ and IgG^+^ atypical MBCs have different levels of somatic hypermutation.

**FIG 1:**
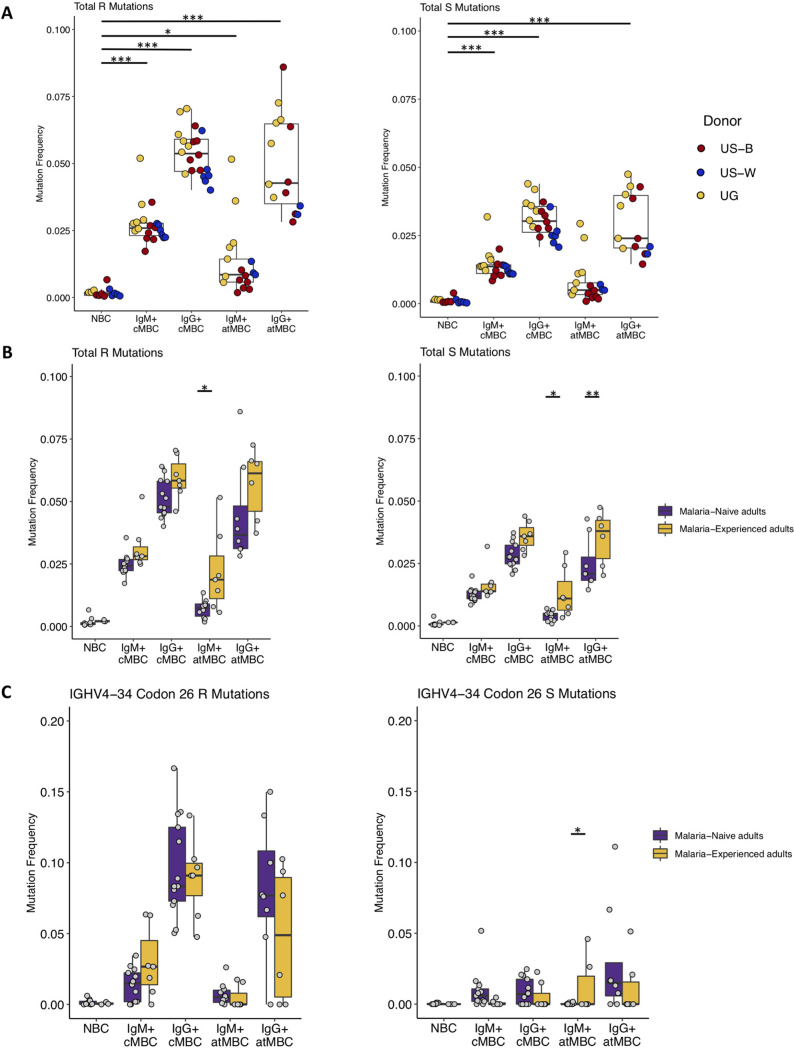
IgM^+^ and IgG^+^ atypical MBCs have different levels of somatic hypermutation. (A) Box plots of the frequency of replacement (R) and silent (S) mutations across the entire V segment in naive B cells (NBCs; *n* = 13), IgM^+^ classical MBCs (cMBC; *n* = 20), IgG^+^ cMBCs (*n* = 20), IgM^+^ atypical MBCs (atMBC; *n* = 17), and IgG^+^ atMBC (*n* = 14). Differences between NBCs and most cell other B cell types are statistically significant, and the exact *P* values are shown in Table S5 at https://doi.org/10.6084/m9.figshare.16449858.v3. US-B, Black U.S. adults; US-W, White U.S. adults; UG, Ugandan adults. (B) Box plots of R and S mutations across the entire V segment comparing malaria-naive (for NBCs, *n* = 10; for IgM^+^ and IgG^+^ cMBCs, *n* = 13; for IgM^+^ atMBCs, *n* = 10; and for IgG^+^ atMBCs, *n* = 8) and malaria-experienced individuals (for NBCs, *n* = 3; for IgM^+^ cMBCs, IgG^+^ cMBCs, and IgM^+^ atMBCs, *n* = 7; and for IgG^+^ atMBCs, *n* = 6). (C) Box plots of R and S mutations in IGHV4-34 codon 26 (IMGT numbering) in malaria-naive (NBCs, *n* = 10; IgM^+^ and IgG^+^ cMBCs, *n* = 13; IgM^+^ atMBCs, *n* = 10; and IgG^+^ atMBCs, *n* = 8) and malaria-experienced (NBCs, *n* = 3; IgM^+^ cMBCs, IgG^+^ cMBCs, and IgM^+^ atMBCs, *n* = 7; and IgG^+^ atMBC, *n* = 6) individuals. For all panels, *, *P* < 0.05; ** *P* < 0.01; and ***, *P* < 0.001 as determined by two-way mixed-measures ANOVA with pairwise comparisons using either Tukey’s test (panel A; *n* = 10) or Šídák's test (panel B and C; *n* = 5).

B cells undergo SHM during the process of affinity maturation in germinal centers. Assessing the levels of SHM in different B cell subtypes can provide insight into the paths taken by these cells during development, contributing to our understanding of the origin of and relation between B cell subtypes in the immune response. To this end, we assessed the frequency of replacement (R) and silent (S) mutations in the IGHV region in NBCs, cMBCs, and atMBCs from malaria-experienced and malaria-naive adults. Regardless of malaria-experience, we observed differences between B cell subsets. As expected, we observed low rates of SHM in NBCs from both malaria-naive and malaria-experienced adults (Fig. 1A). Among cMBCs, the highest average mutation frequencies were observed in the IgG^+^ subset, while IgM^+^ cMBCs showed intermediate levels of SHM, again as expected (Fig. 1A; Table S5 at https://doi.org/10.6084/m9.figshare.16449858.v3). It has been reported that cMBCs and atMBCs in malaria-experienced adults have similar levels of SHM (10, 28). In line with this observation, we observed IgG^+^ atMBCs had similar levels of SHM as IgG^+^ cMBCs (Fig. 1A; Table S5 at the URL mentioned above). However, IgM^+^ atMBCs differed significantly from cMBC subsets. IgM^+^ atMBCs had very low average levels of SHM, with a large percentage of these cells (19% to 83%) having zero R mutations in the V region. This fraction was much lower in IgM^+^ cMBCs (4% to 28%) (Fig. S4 at the URL mentioned above). For one sample, UG-5, we saw higher average levels of SHM, with only 0.36% of IgM^+^ atMBCs and 0.27% of IgM^+^ cMBCs having zero R mutations in the V region. The relative levels of SHM among these B cell populations showed a similar pattern when we divided the IGHV region into CDRs and framework regions (FWRs) (Fig. S5A and B at the URL mentioned above).

To investigate the potential effects of *Plasmodium* exposure on overall SHM levels, we next compared the frequency of R and S mutations between malaria-experienced and malaria-naive adults. Levels of SHM in NBCs and cMBCs were similar between adults regardless of malaria exposure. However, IgM^+^ and IgG^+^ atMBCs from malaria-experienced adults had higher frequencies of S mutations (Fig. 1B). IgM^+^ atMBCs from malaria-experienced adults also had a higher frequency of R mutations than those from malaria-naive adults (Fig. 1B), suggesting exposure to P. falciparum drives increased mutation rates in the atMBC compartment.

Our results are in keeping with models in which IgG^+^ atMBCs have undergone similar levels of antigen-induced activation, affinity maturation, and differentiation and have therefore accumulated similar rates of SHM to cMBCs. For IgM^+^ atMBCs, these results are consistent with extrafollicular activation of NBCs, resulting in few, if any, mutations. Finally, the observation that mutation frequencies between donors were similar across NBC and cMBC populations regardless of malaria experience suggests that SHM rates are intrinsic to these B cell subsets and are not influenced by chronic *Plasmodium* antigen exposure. Conversely, *Plasmodium* exposure seems to drive increased mutation in atMBCs, potentially reflecting increased activation in these cell types in response to chronic antigen exposure.

### Normal selection against autoreactivity in malaria-associated memory B cells.

The IGHV4-34 gene segment is naturally autoreactive in the unmutated state (35, 36). BCRs encoded by this gene segment can be detected using the 9G4 monoclonal antibody. Hart et al. reported that malaria-experienced adults in Mali harbor higher percentages of 9G4^+^ cMBCs and atMBCs than U.S. adults (37). Since mutations in IGHV4-34 codon 26 (ImMunoGeneTics [IMGT] numbering) can reduce autoreactivity (35), the authors also assessed SHM levels in IGHV4-34 in adults from Mali compared to other V genes in the same individuals but did not find any differences regardless of cell population or isotype assessed (37). Here, we specifically compared the rate of SHM in IGHV4-34 codon 26 between malaria-naive and malaria-experienced individuals in an attempt to explain the initial observation by Hart et al. The rate of R mutations varied by cell type, in line with the patterns observed for mutations across the entire V gene, but was similar between malaria-experienced and malaria-naive individuals (Fig. 1C). Our results are thus in line with the prior analysis of mutation levels in IGHV4-34 in malaria-experienced individuals (37). This is in contrast with other reports that lower mutation rates in IGHV4-34 codon 26 were seen in HIV-infected individuals and severe COVID-19 patients than uninfected individuals or patients with mild COVID-19, respectively (21, 27). These results suggest chronic *Plasmodium* exposure, perhaps unlike other chronic or highly inflammatory conditions, does not influence the development of B cells expressing the inherently autoreactive IGHV4-34 gene segment.

### HCDR3 physicochemical properties of IgM^+^ atypical MBCs are similar to those of NBCs.

**FIG 2:**
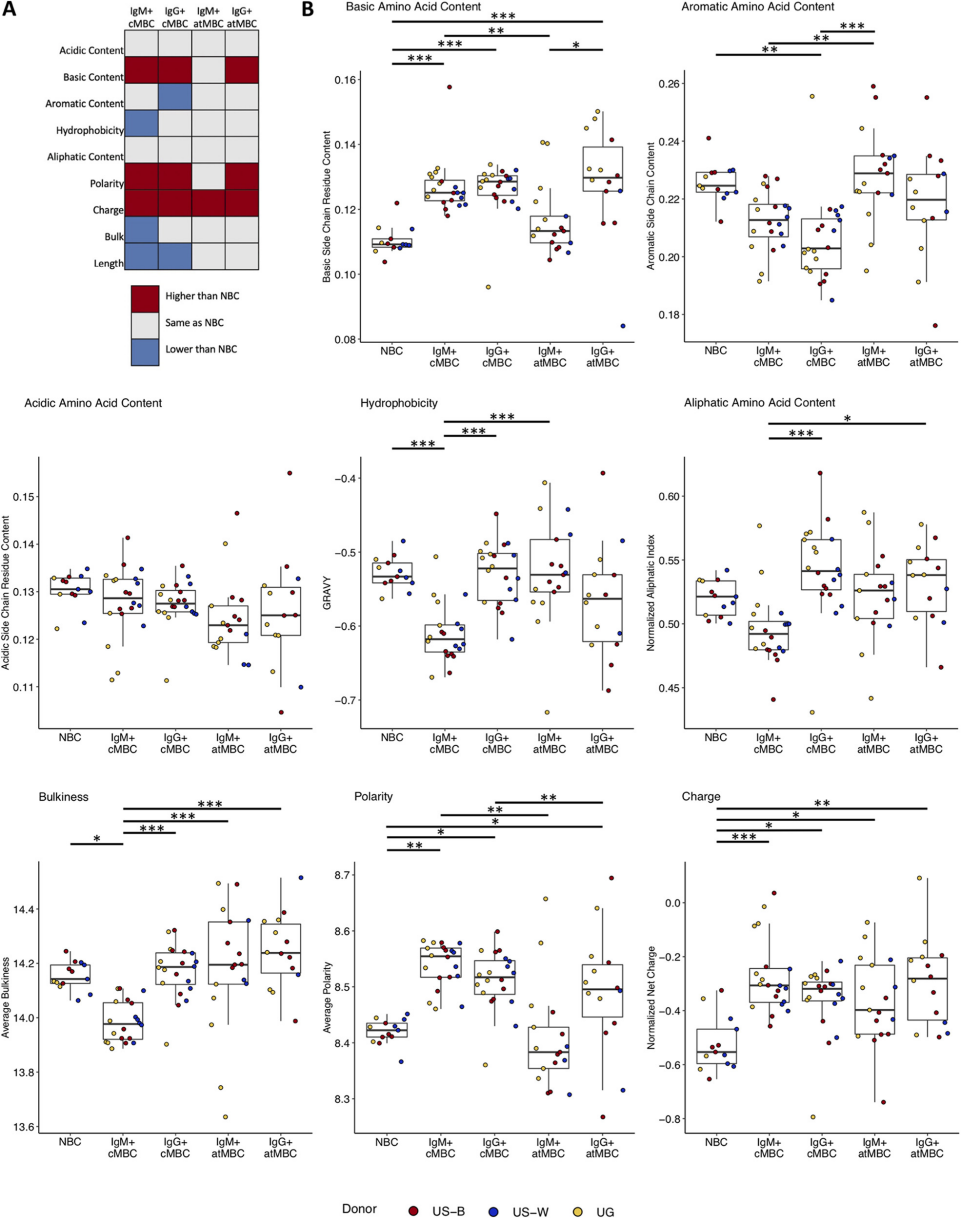
HCDR3 physicochemical properties of IgM+ atypical MBCs are similar to those of NBCs. (A) Summary of differences in HCDR3 physicochemical properties in memory B cell subsets in comparison to naive B cells (NBCs). *P* values are reported in Table S6 at https://doi.org/10.6084/m9.figshare.16449858.v3. (B) Box plots of 8 different HCDR3 physicochemical properties in NBCs (*n* = 13), IgM^+^ classical MBCs (cMBC; *n* = 20), IgG^+^ cMBC (*n* = 20), IgM^+^ atypical MBCs (atMBC; *n* = 17), and IgG^+^ atMBC (*n* = 14). *, *P* < 0.05; **, *P* < 0.01; ***, *P* < 0.001 as determined by two-way mixed-measures ANOVA with pairwise comparisons by Tukey’s *post hoc* test, corrected for multiple comparisons (*n* = 10). US-B, Black U.S. adults; US-W, White U.S. adults; UG, Ugandan adults.

The HCDR3 region is highly variable in amino acid sequence and length between B cells and is an important determinant of antigen binding and specificity. As such, differences in HCDR3 properties between B cell subtypes could be reflective of differences in antigen selection or other selective drivers during development. We therefore investigated differences in the physicochemical properties of this region in different B cell subsets. Prominent differences were seen between B cell subtypes, regardless of exposure to *Plasmodium* antigens, suggesting most physicochemical properties are features of cell type rather than the result of selection by antigen (Fig. 2A).

We first assessed the frequency of amino acids with certain side chain properties in the HCDR3 region. Lower proportions of basic amino acids have previously been reported in NBCs than cMBCs and atMBCs (38). In our data, we saw the same trend, with NBCs having the lowest frequency of basic amino acids (Fig. 2B). IgM^+^ atMBCs were similar to NBCs, while all other cell types contained a higher frequency of basic residues (Fig. 2B). The aromatic base content did not differ between NBCs, IgM^+^ cMBCs, IgM^+^ atMBCs, and IgG^+^ atMBCs, while significant differences were observed between IgG^+^ cMBCs and IgM^+^ atMBCs (Fig. 2B). Finally, the acidic amino acid content was similar between all B cell subsets (Fig. 2B).

We next determined the hydrophobicity of the HCDR3 region by calculating a grand average of hydropathicity index (GRAVY) and aliphatic index (a measure of the relative volume of the HCDR3 region occupied by aliphatic side chains) for each cell type. Again, NBCs and IgM^+^ atMBCs displayed similar GRAVY values and aliphatic indexes, while IgM^+^ cMBCs were most divergent from other B cell populations (Fig. 2B). A similar pattern was observed for bulkiness, a measure of the number of amino acids with bulky side chains (tryptophan, serine, threonine, etc.). The pattern for HCDR3 region polarity was similar to that of the frequency of basic amino acid residues, with NBCs and IgM^+^ atMBCs harboring lower polarity than other cell types (Fig. 2B). Finally, the HCDR3 region charge was higher in all B cell subsets than NBCs (Fig. 2B).

Although some of the differences in physicochemical properties between B cell subsets may appear small, their patterns are consistent across multiple properties, suggesting they have biological relevance and are evidence of differences in the environmental pressures on B cell selection and differentiation. In summary, while the HCDR3 physicochemical properties of IgM^+^ cMBCs are distinct from NBCs, those of IgM^+^ atMBCs are highly similar, indicative of a difference in development and selection between these two IgM^+^ B cell subsets, with IgM^+^ atMBCs being more closely related to NBCs. IgG^+^ atMBCs, on the other hand, show signs of antigen selection similar to IgG^+^ cMBCs.

To determine if exposure to *Plasmodium* antigens influenced HCDR3 physicochemical properties within different B cell subtypes, we assessed differences in physicochemical properties between malaria-naive and malaria-experienced individuals. For all properties assessed, there were no significant differences between malaria-experienced and malaria-naive individuals in NBCs and cMBCs (Fig. S6 at https://doi.org/10.6084/m9.figshare.16449858.v3). Conversely, differences were observed in atMBCs for specific properties. IgM^+^ atMBCs showed increased basic amino acid content, increased charge, and decreased bulkiness in malaria-experienced individuals compared to malaria-naive individuals (Fig. S6 at the URL mentioned above). Additionally, IgG^+^ atMBCs showed increased basic amino acid content in malaria-experienced individuals (Fig. S6 at the URL given above). Together, these data indicate that while physicochemical properties are largely cell type intrinsic, chronic *Plasmodium* antigen exposure may drive selection of specific HCDR3 properties in atMBCs, suggesting that developmental pathways or effects of antigen exposure on bulk BCR repertoires are different between atMBCs and cMBCs.

### Malaria-associated IgG^+^ atypical MBCs have shorter HCDR3 regions than IgG^+^ atypical MBCs from malaria-naive adults.

**FIG 3:**
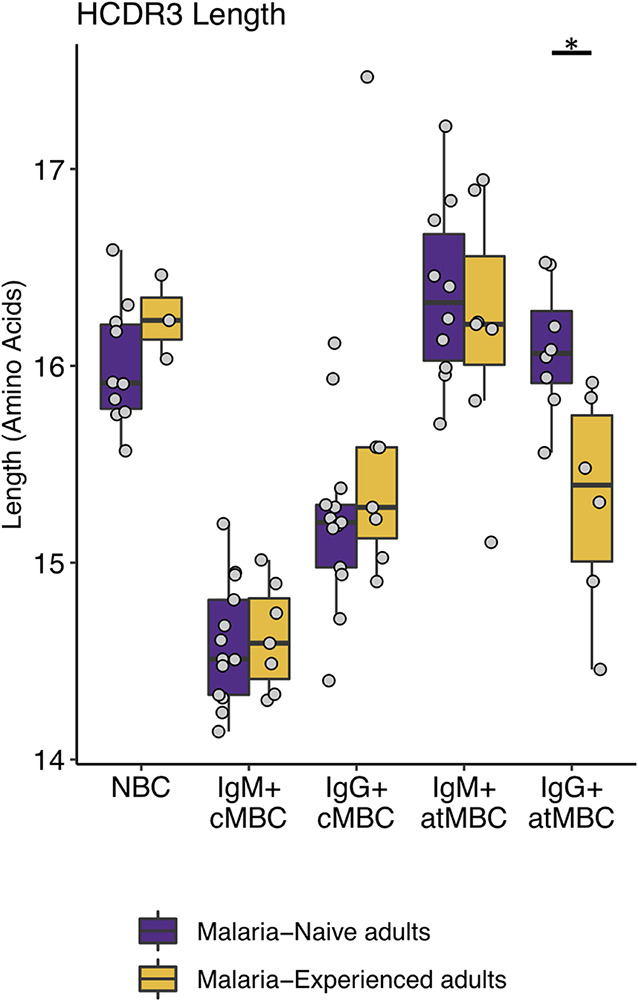
Malaria-associated IgG^+^ atMBCs have shorter HCDR3 sequences. Box plots comparing HCDR3 length in various B cell subsets between malaria-naive (for NBCs, *n* = 10; for IgM^+^ and IgG^+^ cMBCs, *n* = 13; for IgM^+^ atMBCs, *n* = 10; and for IgG^+^ atMBCs, *n* = 8) and malaria-experienced (for NBCs, *n* = 3; for IgM^+^ cMBCs, IgG^+^ cMBCs, and IgM^+^ atMBCs, *n* = 7; for IgG^+^ atMBCs, *n* = 14) individuals. *, *P* < 0.05 as determined by two-way mixed-measures ANOVA with pairwise comparisons using Šídák's test (*n* = 5).

The length of the HCDR3 region affects the ability of a BCR to recognize certain antigens. For example, broadly neutralizing antibodies against HIV often have long HCDR3 regions that are thought to contribute to the recognition of structurally occluded epitopes on viral proteins (27, 39, 40). Although it is not known whether longer HCDR3 regions provide an advantage for the antibody response against P. falciparum, we investigated whether lifelong P. falciparum exposure results in differences in HCDR3 sequence length between malaria-experienced and malaria-naive individuals. It has previously been reported that NBCs generally have longer HCDR3 regions than memory populations (41), which was also observed in our data (Fig. 3). As expected, the HCDR3 length in NBCs was not affected by P. falciparum exposure. Compared to NBCs, IgM^+^ and IgG^+^ cMBC HCDR3 sequences were shorter, and no significant differences were observed between malaria-naive and malaria-experienced adults in these two cell populations. IgM^+^ atMBCs from both groups had HCDR3 sequences of similar length to NBCs. However, IgG^+^ atMBCs showed shorter HCDR3 regions in malaria-experienced individuals (15.3 amino acids [aa]) than in malaria-naive individuals (16.1 aa). These results suggest that the inflammatory environment or chronic antigen exposure of P. falciparum infections may select for shorter HCDR3 regions in the IgG^+^ atMBC subset.

### IGHV gene usage differs between B cell subtypes.

IGHV gene usage has been used to explore differences in developmental pathways between B cell subsets. For example, it is well-known that IGHV gene usage differs between IgM^+^ and IgG^+^ cMBCs (38). In addition, the B cell repertoire is shaped by infection (42) and aging (43). In the case of malaria, antibody responses against circumsporozoite protein (CSP) following vaccination with P. falciparum sporozoites were dominated by IGHV3-30 and IGHV3-33 (44, 45), suggesting that parasite exposure could promote the usage of specific IGHV genes. To explore the IGHV gene usage in atMBCs, we determined IGHV gene frequencies in NBCs, cMBCs, and atMBCs from malaria-naive and malaria-experienced adults.

**FIG 4:**
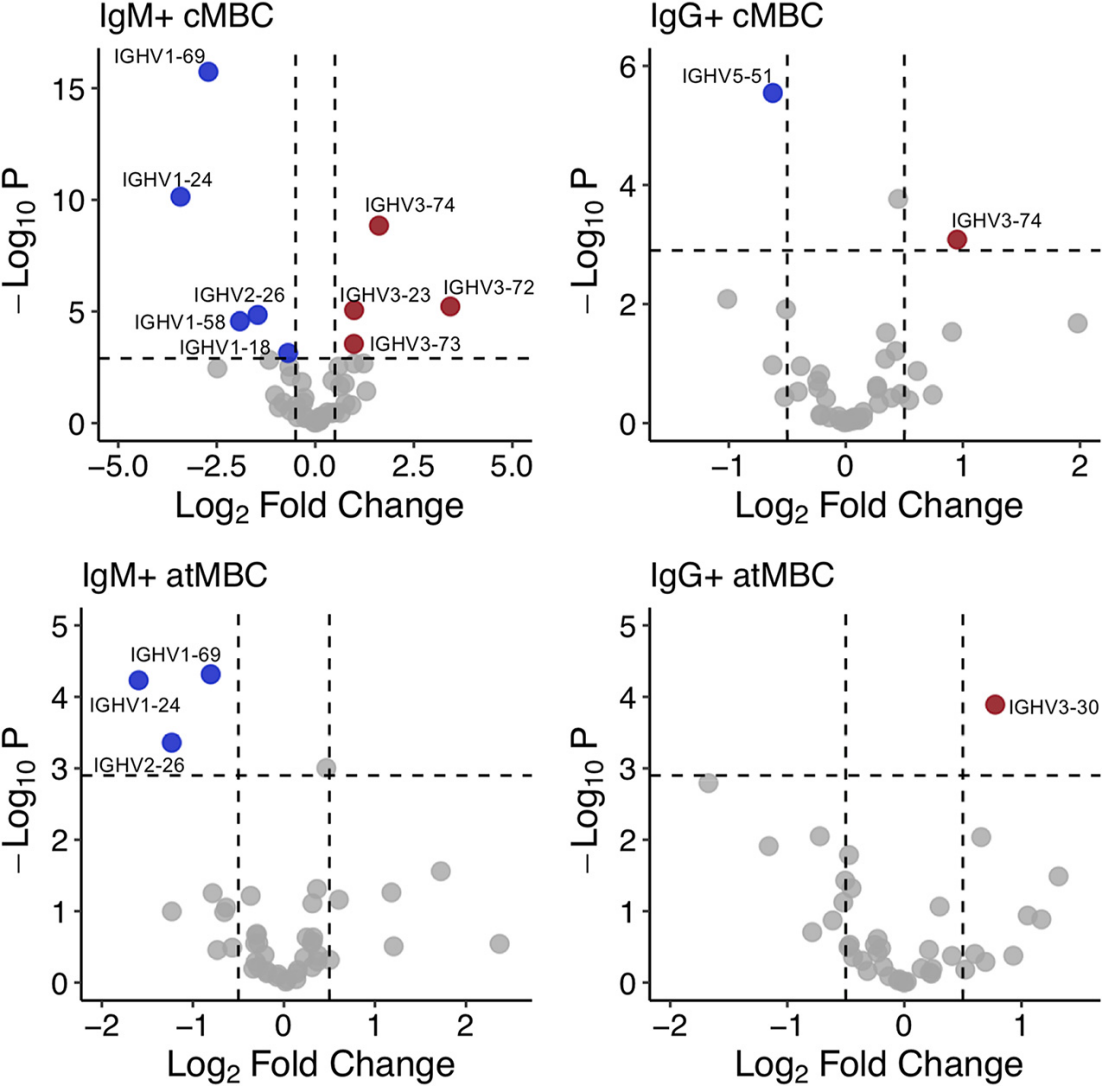
IGHV gene usage differs by cell type. Volcano plots depicting differences in the expression frequency of IGHV genes between naive B cells (NBCs) and other B cell subtypes. Usage is plotted as the log_2_ ratio between IGHV gene usage in IgM^+^ classical MBCs (cMBCs; *n* = 20), IgG^+^ cMBCs (*n* = 20), IgM^+^ atypical MBCs (atMBCs; *n* = 17), or IgG^+^ atMBCs (*n* = 14) and NBCs (*n* = 13). Genes with significantly higher usage in the indicated cell type than in NBCs are marked in red, while genes with significantly lower usage are marked in blue. Statistical significance was determined by using a Student's *t* test with Bonferroni correction for multiple testing (*n* = 44). Dashed horizontal lines indicate corrected *P* value of 0.05. Dashed vertical lines indicate log_2_ fold change of −0.05 and 0.05. Complete lists of IGHV genes with differential usage in MBC subtypes and their *P* values are included in Table S10 at https://doi.org/10.6084/m9.figshare.16449858.v3.

Our data set shares characteristics with previously reported BCR repertoires. IGHV gene usage among NBCs was similar among all individuals (Fig. S3A at https://doi.org/10.6084/m9.figshare.16449858.v3), with expected differences in usage due to ethnicity seen in IGHV4-38-2 and IGHV1-69-2 (46). Previous studies have reported IGHV1-69, IGHV3-23, IGHV3-30, and IGHV4-34 to be among the most commonly used IGHV genes in NBCs, which was also reflected in our data (41, 47, 48) (Fig. S3A at the URL mentioned above). Compared to NBCs, IgM^+^ cMBC showed consistent changes in IGHV gene usage, with several genes used more frequently (IGHV3-23, IGHV3-72, IGHV3-73, and IGHV3-74) or less frequently (IGHV1-18, IGHV1-24, IGHV1-58, IGHV1-69, and IGHV2-26) in IgM^+^ cMBCs regardless of malaria experience (Fig. 4; Fig. S3A at the URL mentioned above). On the other hand, there were fewer consistent changes in IGHV gene usage across individuals in the IgG^+^ cMBC repertoires, which is likely due to unique immunological histories (49). IGHV5-51was the only gene used at a lower frequency by IgG^+^ cMBCs than by NBCs, while IGHV3-74 was used more frequently (Fig. 4; Fig. S3A at the URL given above). Unlike IgM^+^ cMBCs, the BCR repertoire of IgM^+^ atMBCs showed more variation between individuals, with a decrease in the usage of IGHV1-24, IGHV1-69, and IGHV2-26 compared to NBCs being the only consistent changes (Fig. 4; Fig. S3A at the URL mentioned above). In IgG^+^ atMBCs, IGHV3-30 was used at a higher frequency than in NBCs (Fig. 4; Fig. S3A at the URL mentioned above). Some consistent changes in IGHV gene usage, such as the reduced frequency of IGHV2-26 in IgM^+^ cMBCs and atMBCs compared to NBCs, suggest that common processes influence the development or selection of multiple B cell subtypes. The similarities in IGHV gene repertoire among IgM^+^ cMBCs suggest that stimuli other than antigen selection are involved in the differentiation of these cells, while the differentiation of all other B cell subsets examined here is predominantly driven by antigen recognition.

### Higher usage of IGHV3-73 in malaria-experienced individuals.

**FIG 5:**
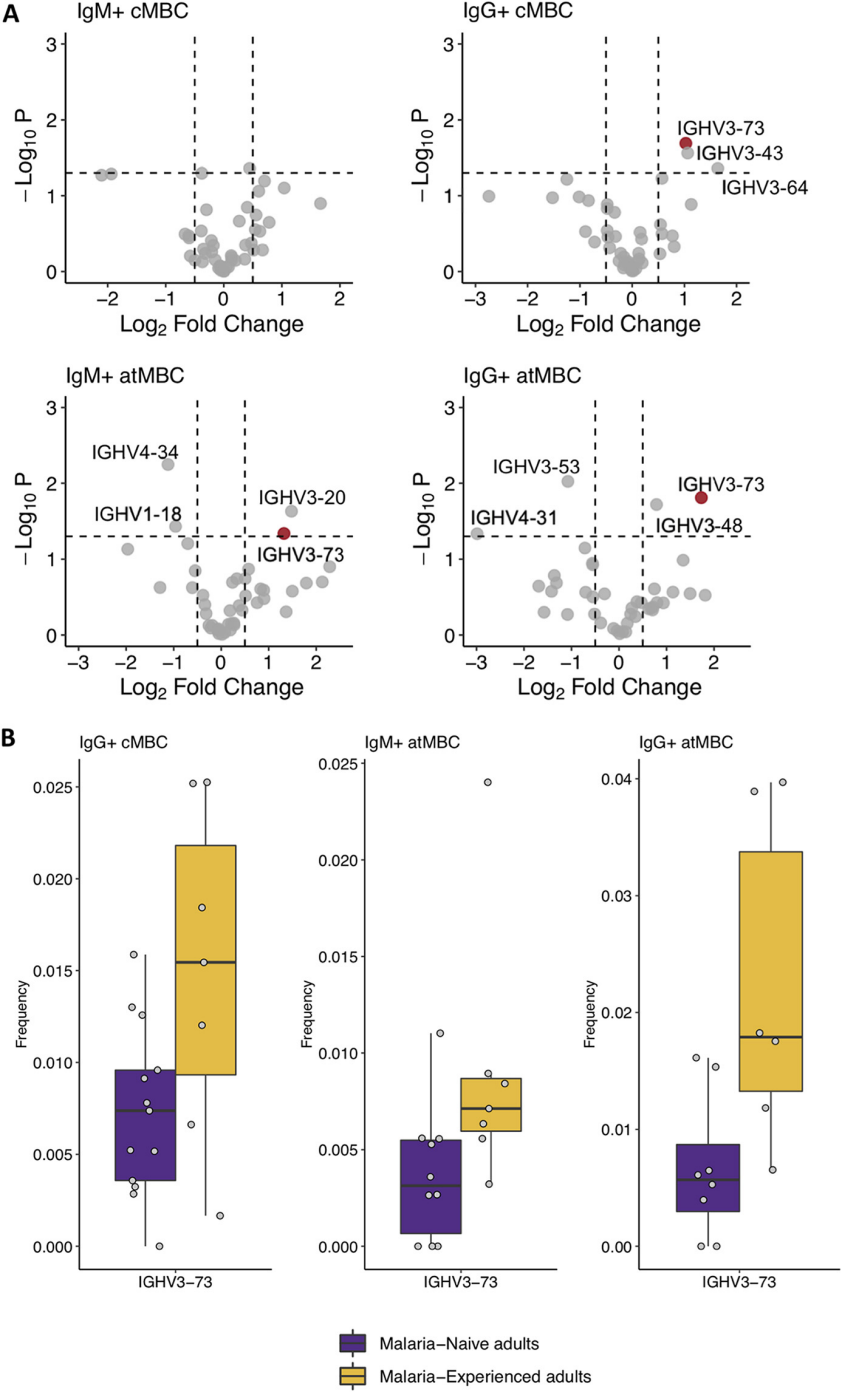
P. falciparum exposure influences IGHV gene usage. (A) Volcano plots of IGHV gene usage in IgM^+^ classical MBCs (cMBCs), IgG^+^ cMBCs, IgM^+^ atypical MBCs (atMBCs), and IgG^+^ atMBCs. Usage is plotted as the log_2_ of the difference between IGHV gene usage in malaria-experienced (IgM^+^ and IgG^+^ cMBCs, *n* = 13; IgM^+^ atMBCs, *n* = 10; and IgG^+^ atMBCs, *n* = 8) and malaria-naive (IgM^+^ and IgG^+^ cMBCs, *n* = 7; IgM^+^ atMBCs, *n* = 7; and IgG^+^ atMBCs, *n* = 6) donors. Dashed vertical lines indicate cutoff log_2_ fold change values of −0.05 and 0.05. Dashed horizontal lines indicate an uncorrected *P* value of 0.05. When corrected for multiple testing (*n* = 44), no differences for individual B cell subsets were statistically significant. IGHV3-73 is highlighted in red. (B) Box plots comparing frequency of IGHV3-73 gene expression between malaria-naive (IgM^+^ and IgG^+^ cMBCs, *n* = 13; IgM^+^ atMBCs, *n* = 10; and IgG^+^ atMBCs, *n* = 8) and malaria-experienced (IgM^+^ and IgG^+^ cMBCs, *n* = 7; IgM^+^ atMBCs, *n* = 7; and IgG^+^ atMBCs, *n* = 6) groups in IgG^+^ cMBCs, IgM^+^ atMBCs, and IgG^+^ atMBCs.

We next compared the relative usage of IGHV genes in cMBCs and atMBCs between malaria-naive and malaria-experienced donors. In IgM^+^ cMBCs, no differences in IGHV gene usage between the groups were observed (Fig. 5), in line with our observation that IGHV gene usage in this B cell subset is, at least in part, driven by other factors than antigen selection. In IgG^+^ cMBCs, IgM^+^ atMBCs, and IgG^+^ atMBCs, several IGHV genes showed differential usage between malaria-naive and malaria-experienced individuals (Fig. 5; Fig. S7 at https://doi.org/10.6084/m9.figshare.16449858.v3). When analyzing IGHV gene usage, we tested for statistical significance using Student’s *t* tests. After correcting for multiple testing (*n* = 44), none of the observed differences between malaria-experienced and malaria-naive individuals were statistically significant. Uncorrected *P* values are reported in Table S10 at the URL mentioned above. However, IGHV3-73 was used more frequently in malaria-experienced individuals in all three MBC subsets (Fig. 5B). While increased usage of this IGHV gene in each individual subset was not statistically significant, the chance that this gene would be randomly included in the list of differentially expressed genes in all three cell types is extremely small. B cells expressing IGHV3-73 may thus have a biological significance in the context of *Plasmodium* infections. These results suggest that *Plasmodium* antigen exposure may drive the usage of certain IGHV genes in MBCs.

## DISCUSSION

In this study, we assessed how P. falciparum exposure shapes the B cell compartment by characterizing the BCR repertoire of 13 malaria-naive and 7 malaria-experienced adults. In particular, we were interested in answering the question of whether BCR properties of atMBCs differ between P. falciparum-naive and P. falciparum-exposed individuals to better understand the developmental pathways of atMBCs in these different settings. Overall, we show that exposure to *Plasmodium* infection drives changes to HCDR3 length, the usage of a specific IGHV gene, and SHM levels in atMBCs, while differences in amino acid properties between B cell populations are more likely to be cell intrinsic.

Studies on atMBCs in individuals from malaria regions of endemicity have largely focused on IgG^+^ atMBCs or have considered IgM^+^ and IgG^+^ atMBCs as a single population. In this study, we considered these populations separately to assess the atMBC subcompartments in more depth. We found that BCR properties of IgM^+^ atMBCs and IgG^+^ atMBCs are distinct. IgM^+^ atMBCs display low levels of SHM, more similar to rates seen in NBCs than in IgG^+^ atMBCs or cMBCs. IgM^+^ atMBCs also resemble NBCs in HCDR3 physicochemical properties, with no statistically significant differences observed between NBCs and IgM^+^ atMBCs for all but one property assessed, including HCDR3 sequence length. In contrast, IgG^+^ atMBCs displayed similar trends as IgG^+^ cMBCs in HCDR3 physicochemical properties and SHM rates. The differences in BCR characteristics between IgM^+^ and IgG^+^ atMBC subsets suggest that these cells follow different developmental pathways. Based on the many similarities in BCR repertoire between IgM^+^ atMBCs and NBCs, we propose that IgM^+^ atMBCs represent a population of extrafollicularly activated NBCs, which would account for the low levels of SHM seen in this population. As such, these cells could be closely related to activated NBCs in systemic lupus erythematosus (SLE) patients, which also lacked SHM and gave rise to auto-antibody-secreting cells (25). Conversely, higher levels of SHM in IgG^+^ atMBCs than IgM^+^ atMBCs suggest that, similar to cMBCs, IgG^+^ atMBCs pass through the germinal center and undergo rounds of SHM and affinity maturation, although extrafollicular affinity maturation has also been reported (50). This is in line with recent reports that atMBCs may be a normal component of the immune response (51, 52) and contrasts with the proposed extrafollicular origin of phenotypically similar DN2 cells in SLE patients (19). Additionally, we observed no evidence of altered selection against autoreactivity in IGHV4-34 in malaria-experienced individuals, in line with what has been previously reported (37). This result contrasts with studies in HIV-infected individuals and severe COVID-19 patients that have shown decreased mutation levels in IGHV4-34 (21, 27). Together, differences in SHM levels between IgG^+^ atMBCs in malaria-experienced individuals and phenotypically similar cells found in other conditions suggest that the immune responses stimulated by P. falciparum infection and other highly inflammatory environments differ. In addition, because of the many differences in BCR repertoire and likely differences in developmental pathways between IgM^+^ and IgG^+^ atMBCs, these two cell populations should be considered independently when studying the atypical B cell compartment in the context of malaria.

Interestingly, a recent single-cell transcriptomics study in malaria-naive and malaria-experienced individuals by Sutton et al. showed representation of both IgM^+^ and IgG^+^ atypical B cells in the same clusters, suggesting that these cells have similar transcriptional profiles and may be developmentally related (52). However, these atypical B cell clusters were not comprised exclusively of CD21^−^ CD27^−^ B cells, which complicates the comparison between those results and observations reported here. It is possible that atypical B cells that are not CD21^−^ CD27^−^ are influenced differently by antigen exposure and have different BCR repertoires than the atMBCs included in this study. The differences in BCR physicochemical properties and IGHV gene repertoire between B cell subsets observed here raises the hypothesis that certain BCR characteristics may skew B cells toward a particular fate. If this were the case, little overlap in B cell lineages would be expected between B cell subsets. Similar to the report by Sutton et al., we observed more clonal sharing within subsets than between subsets. This observation suggests that B cell lineages can differentiate into different subsets but may be restricted in their fate by the characteristics of their BCR.

Our results imply that P. falciparum exposure influences the selection and development of atMBCs. We observed increased levels of SHM in IgM^+^ and IgG^+^ atMBCs from malaria-experienced individuals compared to malaria-naive individuals. In keeping with these findings, Hopp et al. showed increased SHM in *Plasmodium*-specific IgG^+^ B cells compared to hemagglutinin-specific IgG^+^ B cells from the same donors (53), and Sutton et al. observed lower levels of SHM in malaria-naive individuals than malaria-experienced individuals (52). Together, these data suggest that *Plasmodium* exposure drives higher levels of SHM in *Plasmodium*-specific B cells. In addition to increased mutation rates, IgM^+^ atMBCs from malaria-experienced adults showed differences in specific physicochemical properties of the HCDR3 region compared to IgM^+^ atMBCs from malaria-naive adults, suggesting that antigen exposure may drive differences in development or selection of these cells. Finally, atMBCs from malaria-experienced individuals utilized specific IGHV genes at different frequencies than atMBCs from malaria-naive individuals. The most prominent difference was observed for IGHV3-73, which was used more frequently in IgG^+^ cMBCs, IgM^+^ atMBCs, and IgG^+^ atMBCs from malaria-experienced individuals. Investigating the antigen specificity of IGHV3-73-using B cells in malaria-experienced individuals may help uncover what promotes the usage of this IGHV gene. Sutton et al. recently reported that they did not find a difference in IGHV gene usage between malaria-experienced and malaria-naive individuals; however, in this study, only two malaria-experienced individuals were compared to two malaria-naive individuals (52). The various differences in BCR repertoire between atMBCs from malaria-experienced and malaria-naive adults suggest that atMBCs may be influenced by chronic antigen exposure.

It has previously been reported that IgG^+^ cMBCs and IgG^+^ atMBCs from malaria-experienced adults had similar HCDR3 sequence lengths (28). Our data are in alignment with these results, and we report similar average HCDR3 lengths in malaria-associated IgG^+^ atMBCs as previously published (15.3 aa in our study versus 15.74 aa in Zinocker et al.) (28). However, we observed that HCDR3s in IgG^+^ atMBCs from malaria-naive adults, on average, are longer than those in IgG^+^ atMBCs from malaria-experienced adults. One explanation for this observation could be that P. falciparum infection drives the selection of BCRs with shorter HCDR3 sequences in IgG^+^ atMBCs. *In vitro* stimulation by TLR9, TLR7/IFN-α, or TLR9/IFN-α was shown to decrease HCDR3 length in B cells (54). Since *Plasmodium* DNA can serve as a TLR9 ligand (55), we hypothesize that the shorter HCDR3 sequences seen in IgG^+^ atMBCs may be the result of TLR9 stimulation. Alternatively, influenza virus infections or other viral infections may select for longer HCDR3s in the IgG^+^ atMBC subset in malaria-naive individuals, resulting in enrichment of this subset for B cells with specificity for occluded targets, such as epitopes on the conserved influenza A virus hemagglutinin stem region (56), in contrast to enrichment for atMBCs with specificity for *Plasmodium* antigens in malaria-experienced individuals.

Our study has several limitations. First, the number of individuals we included in this study was relatively small, with 13 malaria-naive and 7 malaria-experienced donors. Additionally, our data set is not ultradeep. We did not sort cMBCs and atMBCs by isotype but instead separated IgM^+^ and IgG^+^ B cells by sequence during our downstream analysis, which resulted in an overrepresentation of IgM sequences. While the relatively small number of donors and limited sequencing depth reduced our power to detect differences between B cell subsets, we were able to reproduce results reported by others. We observed low levels of SHM in NBCs, while the highest levels of SHM were seen in IgG^+^ cMBCs, as previously published (41). IGHV genes known to be common in NBCs, including IGHV1-69 and IGHV3-23, were found highly expressed in the NBC subset. Previously reported differences in IGHV gene usage due to race were also recapitulated in our study (46). Additionally, differences in HCDR3 physicochemical properties between B cell subsets in our data set were consistent with another report (38). For example, IgM^+^ cMBCs are known to have a lower hydrophobic index and lower aliphatic index than other B cell types, which was also observed in our data. Similarities between our data set and data sets from others give us confidence that the differences we observed between B cell subsets and between malaria-naive and malaria-experienced individuals are true differences that can be extrapolated to a larger population. Finally, this study assessed the entire BCR repertoire without taking into account antigen specificity, which could explain discrepancies between our study and others. For example, Muellenbeck et al. reported higher average levels of SHM in *Plasmodium*-specific IgG^+^ atMBCs than cMBCs (11). In contrast, we observed similar SHM rates in bulk IgG^+^ cMBCs and IgG^+^ atMBCs. P. falciparum*-*specific atMBCs may be subject to higher rates of SHM. Recently, Horns et al. showed that, following an influenza infection, bystander-activated cells that were not specific for influenza antigens made up a significant portion of atMBC-like CD11c^+^ T-bet^+^ B cells (57). By assessing all atMBCs, we may have included bystander-activated, nonspecific B cells that are expected to undergo little to no SHM, as well as B cells with specificity for self-antigens such as phosphatidylserine (58). This could explain the lower levels of SHM we report for these cells. While our results suggest that some differences in BCR repertoire are due to malaria experience, by not assessing *Plasmodium*-specific B cells, we cannot completely rule out the possibility that other differences in environment between malaria-naive (U.S. donors) and malaria-experienced (Ugandan donors) individuals have also contributed.

Overall, in this study, we have shown differences in BCR repertoire between malaria-naive and malaria-experienced individuals, most notably differences in IGVH gene usage, HCDR3 length, and level of SHM in atMBCs (summarized in Table 1). Striking differences between IgM^+^ and IgG^+^ atMBCs regardless of malaria experience demonstrate that these populations may follow different developmental pathways during the immune response and should be considered separately in future studies of the B cell response to *Plasmodium* infection.

**TABLE 1:** Summary of key findings

Property	Results	Conclusions
Somatic hypermutation (SHM)	IgM^+^ atMBCs have very low SHM levels, IgG^+^ atMBCs have similar SHM levels as IgG^+^ cMBCs, and increased levels of SHM in atMBCs in malaria-experienced individuals	IgG^+^ atMBCs undergo similar levels of antigen-induced activation, affinity maturation, and differentiation as IgG^+^ cMBCs, while IgM^+^ atMBCs are likely a population of extrafollicularly activated cells. P. falciparum exposure may drive increased mutation rates in atMBCs.
HCDR3 physiochemical properties	IgM^+^ atMBCs largely resemble NBCs, some properties of IgM^+^ atMBCs were influenced by malaria-experience, and malaria-associated IgG^+^ atMBCs have shorter HCDR3 lengths.	Physiochemical properties of the HCDR3 region are largely cell intrinsic and suggest that IgM^+^ atMBCs are more closely related to NBCs than other MBC populations. P. falciparum exposure seems to select for specific properties in atMBCs, particularly shorter HCDR3s in IgG^+^ atMBCs.
IGHV gene usage	Consistent changes in IGHV gene usage seen in IgG^+^ cMBCs, IgM^+^ atMBCs, and IgG^+^ atMBCs. IGHV3-73 used more frequently in IgG^+^ cMBCs, IgM^+^ atMBCs, and IgG^+^ atMBCs	Common processes may influence the selection or development of multiple B cell subtypes while P. falciparum exposure seems to promote the usage of specific IGHV genes, particularly IGHV3-73.

## MATERIALS AND METHODS

### PBMC isolation.

PBMCs from U.S. blood donors were isolated from buffy coats obtained the day following blood draws from Interstate Blood Bank (Memphis, TN). Donor characteristics are summarized in Table S1 at https://doi.org/10.6084/m9.figshare.16449858.v3. Buffy coats were stored and shipped at room temperature (RT) and processed immediately upon arrival. Cells were diluted approximately 4 times in phosphate-buffered saline (PBS) with 2 mM EDTA. PBMCs were layered on Ficoll-Paque (GE Healthcare) and spun at 760 × *g* for 20 min at RT to pellet erythrocytes and separate leukocytes. The leukocytes were then washed in PBS with 2 mM EDTA and centrifuged at 425 × *g* for 15 min at RT, followed by centrifugation at 250 × *g* for 10 min at RT for all subsequent wash steps necessary to remove all platelets. Next, PBMCs were resuspended in Iscove’s modified Dulbecco’s medium (IMDM)/GlutaMAX (Gibco) supplemented with 10% heat-inactivated fetal bovine serum (FBS) and 5% cell culture-grade dimethyl sulfoxide (DMSO), counted, and cryopreserved in liquid nitrogen vapor at a concentration of 20 to 30 million cells per ml. PBMCs from Ugandan donors were isolated according to the same protocol, cryopreserved in Uganda on the day of isolation, and shipped to the United States in a liquid nitrogen transfer vessel.

### B cell isolation.

Cryopreserved PBMCs were thawed in a 37°C water bath and immediately mixed with prewarmed thawing medium (IMDM/GlutaMAX supplemented with 10% heat-inactivated FBS and 0.01% Universal Nuclease [Thermo; catalog no. 88700]). Cells were then centrifuged (250 × *g*, 5 min at RT). The cell pellet was resuspended in thawing medium, and viable cells were counted using trypan blue on a Cellometer Mini (Nexcelom) automated cell counter. Next, cells were pelleted by centrifugation (250 × *g*, 5 min at RT) and resuspended in isolation buffer (PBS supplemented with 2% heat-inactivated FBS and 1 mM EDTA) at 50 million live cells per ml and filtered through a 35-μm sterile filter cap (Corning; catalog no. 352235) to break apart aggregated cells. B cells were isolated using the EasySep human B cell isolation kit (StemCell; catalog no. 17954) according to the manufacturer’s instructions. After washing with PBS, the isolated B cells were incubated with 1 μl per 1 ml LIVE/DEAD Fixable Aqua dead cell stain kit (Thermo Fisher Scientific; catalog no. L34965) for 30 min on ice. Cells were washed with cold PBS with 1% bovine serum albumin (BSA) and then incubated at 4°C for 30 min with an antibody cocktail to label B cell surface markers (Table S8 at https://doi.org/10.6084/m9.figshare.16449858.v3). Before acquisition on a BD FACSAria II cell sorter, the cells were washed with cold PBS with 1% BSA, pelleted by centrifuge at 250 × *g* for 5 min at 4°C, diluted to 20 to 30 million cells per ml in PBS with 1% BSA, and filtered into a fluorescence-activated cell sorter (FACS) tube through a 35-μm sterile filter cap. Lymphocytes were gated using forward and sideward scatter, followed by doublet exclusion. Cells were then gated on live cells, followed by CD19^+^ CD20^+^ B cells (US-B, US-W, and UG-1-UG-3) or CD19^+^ (UG-4-UG-7). NBCs, cMBCs, and atMBCs were separated based on CD21 and CD27 expression (Fig. S2 at the URL mentioned above). Cells were sorted into cold PBS with 1% BSA, spun down (250 × *g*, 5 min at 4°C), resuspended in TRI reagent (Zymo; catalog no. R2050-1), and stored at −70°C.

### BCR sequencing.

RNA was isolated from sorted B cells using the Direct-zol RNA microprep kit (Zymo; catalog no. R2062) and eluted in 11 μl. BCR-seq libraries were then generated as previously described (59) with critical modifications. In short, 10 μl RNA was incubated with 5 μl of human IGH cDNA synthesis primer mix, in which each primer was present at a concentration of 10 μM (Table S7 at https://doi.org/10.6084/m9.figshare.16449858.v3) at 65°C for 2 min. The RNA was then immediately reverse transcribed using SMARTscribe Moloney murine leukemia virus reverse transcriptase (TaKaRa; catalog no. 639537) at 5 U/μl in a total volume of 50 μl, template switch oligonucleotide (TSO; 1 μM final concentration [f/c]; IDT-DNA) (Table S7 at the URL mentioned above), deoxynucleoside triphosphates (dNTPs) (f/c of 1 mM each), dithiothreitol (DTT) (4 mM f/c), RNase Out (2 U/μl; Thermo; catalog no. 10777019), and first strand buffer (TaKaRa) for 2 h at 42°C. The TSO was designed with two isodeoxynucleotides at the 5′ end to prevent TSO concatemerization and three riboguanosines at the 3′ end for increased binding affinity to the appended deoxycytidines. Twelve and a half units of uracil DNA glycosylase (NEB; catalog no. M0280) were added to the reaction mixture, followed by a 40-minute incubation at 37°C. cDNA was purified using Zymo’s RNA Clean & Concentrator kit (catalog no. R1016) and their adapted protocol for large RNAs to remove TSOs.

RNA was extracted from 50,000 NBCs or 20,000 cMBCs, and all available atMBCs (4,503 to 26,301 cells), and reverse transcribed. For the first, seminested PCR, the BCR heavy chain variable region was amplified from cDNA by PCR with forward primer “PCR 1 primer” (200 nM f/c) and a human IGH reverse primer mix (200 nM, each f/c) (Table S7 at https://doi.org/10.6084/m9.figshare.16449858.v3). The PCR was carried out with 1.25 units of AccuStart HiFi polymerase (Quantabio; catalog no. 95085), magnesium sulfate (2 mM f/c), dNTPs (200 μM f/c), and HiFi buffer in a total volume of 50 μl using the program of 94°C for 1 min, 18 cycles of 94°C for 20 s, 60°C for 30 s, and 68°C for 40 s, and a final extension at 68°C for 5 min. DNA was purified using 0.7× Ampure XP paramagnetic beads (Beckman Coulter; catalog no. A63880) and eluted in 10 mM Tris, pH 8.0.

The optimal number of PCR cycles for the second PCR was determined by running a test PCR with AccuStart HiFi polymerase using the program 94°C for 1 min, 11 to 17 cycles of 94°C for 20 s, 60°C for 30 s, and 68°C for 40 s, and a final extension at 68°C for 2 min. The PCR product was visualized on an agarose gel, and the final numbers of PCR cycles were chosen such that comparable amounts of DNA from each sample were multiplexed to generate libraries for sequencing. Sample barcodes were added using primer cocktails 1 to 4 (Table S7 at https://doi.org/10.6084/m9.figshare.16449858.v3) during the second PCR. DNA was purified using 0.7× Ampure XP beads and eluted in 10 mM Tris, pH 8.0.

Samples were multiplexed, followed by library preparation using the DNA library prep master mix set for Illumina (NEB; catalog no. E6040). Following adaptor ligation, DNA fragments of approximately 700 bp were selected by dual size selection with Ampure XP beads. DNA was amplified by PCR using NEBNext Multiplex Oligos for Illumina index primers set 1 (NEB; catalog no. E7335) and the following program: 98°C for 30 s, 6 cycles of 98°C for 10 s and 65°C for 75 s, and a final extension at 65°C for 5 min. Libraries were sequenced on the Illumina MiSeq platform using an asymmetric 400 plus 100 paired-end nucleotide run by the UT Health San Antonio Genome Sequencing Facility.

### Repertoire analysis.

Demultiplexing of sequence reads and the generation of consensus sequences for UMI groups was performed as outlined by Turchninova et al. using software tools MIGEC (v1.2.9) and MiTools (v1.5) (59). Reads derived from IgM and IgG heavy chain sequences were then extracted based on unique sequences in the IgM and IgG constant region (CAACCCTTTTCCCCCT for IgM and CATC(C/G)GTCTTCCCCCT for IgG). Isotype-specific files were further analyzed using software available through the ImmCantation Portal, a collection of Python and R packages for preprocessing, population structure determination, and repertoire analysis of BCR and T cell receptor (TCR) repertoires (34).

The ImmCantation package Change-O (v0.4.4) aligns lymphocyte receptor sequences to germline sequences for downstream analyses (34). Isotype-specific data files were converted into standardized tab-delimited database files required for subsequent Change-O modules to operate using IgBLAST (v1.14.0), which is included in Change-O. Clonal groups were generated using the Change-O module for clustering sequences into clonal groups. The threshold for trimming the hierarchical clustering of B cell clones was determined by the SHazaM module (v1.0.0) for determining distance to nearest neighbor (34). This module estimates the optimal distance threshold for dividing clonally related sequences and generates histograms for manual inspection as well as automated threshold detections. When available, the automated threshold was used for clonal grouping. For some samples, there were too few clones for the program to calculate a threshold. In these cases, the threshold was determined by visually inspecting the generated histogram. With the Change-O DefineClones function, clones were assigned based on IGHV genes, IGHJ gene, and junction distance calculated by SHazaM. The clones were output in a clone-pass file that was used for downstream processing with additional ImmCantation modules. Independent clone-pass files were generated for each biological replicate. For downstream analysis, biological replicate clone-pass files were combined.

IGHV gene usage was analyzed using the Alakazam module (v1.0.1) for basic gene usage analysis (34) and the Change-O-generated clone-pass files. Usage frequency was calculated using the default settings for quantification based on clonal grouping.

To calculate SHM frequency, germline sequences were first inferred for our data set using the Change-O module for reconstructing germline sequences. This module reconstructs germline IGH-V(D)J sequences and generates germline-pass files, which were used in downstream analyses. SHM frequency was determined from these germline-pass files using the SHazaM module for mutation analysis (34). The mutation frequency across the entire IGHV regions (denoted as total mutations) for replacement (R) and silent (S) mutations was determined using the regionDefinition argument IGMT_V_BY_SEGMENTS. R and S mutation frequency across CDR1 and CDR2 (CDR mutations), as well as FWR1, FWR2, and FWR3 (FWR mutations), were calculated individually with the regionDefinition IGMT_V argument. Average frequencies for each donor and cell type are reported. Mutation frequency in IGHV4-34 codon 26 was determined using the regionDefinition argument IGMT_V_BY_CODONS, which calculates R and S mutation frequencies for each codon based on the IGMT numbering system.

HCDR3 physicochemical properties, including sequence length, were determined using the Alakazam module for amino acid physicochemical property analysis. Properties were determined using the default arguments. The first codon and last codon were removed from the HCDR3 sequence prior to the analysis.

### Luminex assay.

A multiplex bead array assay to measure anti-*Plasmodium* IgG was performed as previously described (60, 61). Briefly, total IgG responses to three P. falciparum blood-stage antigens (GLURP-R2, AMA1, and MSP1-19) were assayed in plasma at a 1:1,000 dilution. Antigens were coupled to magnetic MagPlex microsphere beads (Luminex Corp.) in the following concentrations: GLURP-R2, 0.017 ng per 5,000 beads; AMA1, 1.54 ng per 5,000 beads; and MSP1-19, 20.6 ng per 5,000 beads. Fifty microliters of pooled antigen-coupled bead suspension were added to each well of a 96-well plate, and the beads were washed with PBS with 0.05% Tween 20 and incubated with 50 μl of diluted test plasma from Ugandan donors. Pooled hyperimmune Ugandan serum was used as a positive control. Serum from malaria-naive European adults was used as a negative control. Beads were incubated for 1.5 h at RT, washed with PBS-Tween 20, and incubated with 1:200 goat anti-human IgG rPE-labeled secondary antibody (Jackson ImmunoResearch Laboratories; catalog no. 109-116-098) diluted in PBS-Tween 20 for 1.5 h at RT with agitation at 500 rpm. The beads were washed with 1× PBS and subsequently read on a MagPix machine (Luminex Corp.).

### Statistics.

To test for statistically significant differences, we used parametric tests since the assumptions of normality and homoscedasticity were not violated. Since there was missing data in a paired analysis, two-way mixed-measures analysis of variance (ANOVA) was used to test for statistical differences in SHM and HCDR3 properties (including HCDR3 length) between B cell subsets and between malaria-experienced and malaria-naive individuals. Šídák's *post hoc* test for comparisons, corrected for multiple testing, was used to determine whether differences between malaria-naive and malaria-experienced individuals were statistically significant. Tukey’s *post hoc* test for pairwise comparisons, corrected for multiple testing, was used to determine which differences between pairs of B cell subsets were statistically significant. Two-way mixed-measures ANOVA and *post hoc* tests were performed using GraphPad Prism (v9.2.0). Student’s *t* tests were used to test for statistically significant differences in IGHV gene usage between B cell subsets and between malaria-naive and malaria-experienced adults. Pearson’s correlation coefficients were calculated to compare IGHV gene profiles between biological replicates. For all tests, a *P* value of less than 0.05 was considered statistically significant.

### Graphs.

All graphs and heatmaps were generated using ggplot2 in R (v3.6.3). In all box plots, the center line indicates the median, upper and lower limits of the box indicate the first and third quartiles, and the whiskers indicate the upper and lower limits of the data within 1.5 times the interquartile range. Dots indicate average value of each individual donor. For all data sets, the average and median were similar.

### Ethics statement.

The PRISM program included three concurrent dynamic cohort studies in settings with various malaria parasite transmission intensities to measure the incidence of malaria and indicators of malaria morbidity (29). Participants enrolled in this study were from the Nagongera subcounty in Tororo District, Uganda, and have provided written consent for the use of their samples for research. The PRISM cohort study was approved by the Makerere University School of Medicine Research and Ethics Committee (SOMREC), London School of Hygiene and Tropical Medicine institutional review board (IRB), and the University of California, San Francisco Human Research Protection Program and IRB. The collection of PBMCs from U.S. blood donors was considered not human research by the Institutional Review Board of the University of Texas Health Science Center at San Antonio.

### Data availability.

All sequencing files are available from the NCBI Sequencing Read Archive under BioProject accession no. PRJNA694159.
